# Charge Transport Across Au–P3HT–Graphene van der Waals Vertical Heterostructures

**DOI:** 10.1021/acsami.2c13148

**Published:** 2022-10-14

**Authors:** Jacopo Oswald, Davide Beretta, Michael Stiefel, Roman Furrer, Alessia Romio, Michel Daher Mansour, Dominique Vuillaume, Michel Calame

**Affiliations:** † Transport at Nanoscale Interfaces Laboratory, EMPA, Swiss Federal Laboratories for Materials Science and Technology, Überlandstrasse 129, DübendorfCH-8600, Switzerland; ‡ Swiss Nanoscience Institute, University of Basel, Klingelbergstrasse 82, BaselCH-4056, Switzerland; § Department of Physics, University of Basel, Klingelbergstrasse 82, BaselCH-4056, Switzerland; ∥ Institute of Electronic, Microelectronic and Nanotechnology, Centre National de la Recherche Scientifique, Villeneuve d’Ascq59652, France

**Keywords:** organic, semiconductor, graphene, interface, transport, vertical, van der Waals

## Abstract

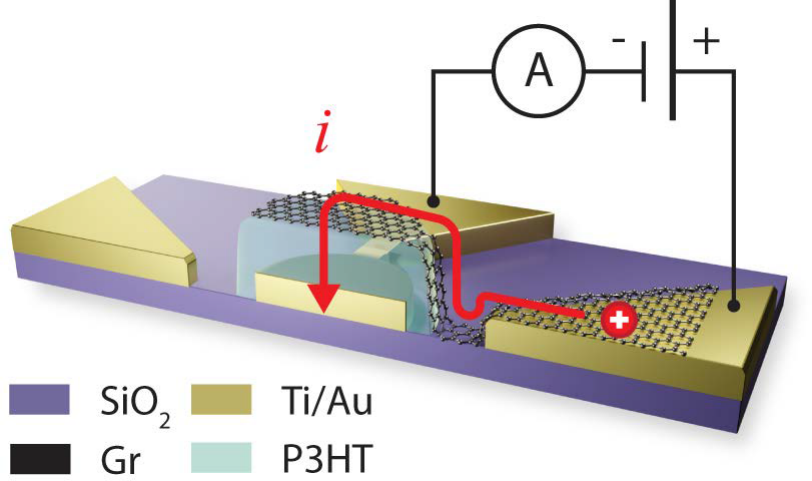

Hybrid van der Waals heterostructures based on 2D materials and/or organic thin films are being evaluated as potential functional devices for a variety of applications. In this context, the graphene/organic semiconductor (Gr/OSC) heterostructure could represent the core element to build future vertical organic transistors based on two back-to-back Gr/OSC diodes sharing a common graphene sheet, which functions as the base electrode. However, the assessment of the Gr/OSC potential still requires a deeper understanding of the charge carrier transport across the interface as well as the development of wafer-scale fabrication methods. This work investigates the charge injection and transport across Au/OSC/Gr vertical heterostructures, focusing on poly(3-hexylthiophen-2,5-diyl) as the OSC, where the PMMA-free graphene layer functions as the top electrode. The structures are fabricated using a combination of processes widely exploited in semiconductor manufacturing and therefore are suited for industrial upscaling. Temperature-dependent current–voltage measurements and impedance spectroscopy show that the charge transport across both device interfaces is injection-limited by thermionic emission at high bias, while it is space charge limited at low bias, and that the P3HT can be assumed fully depleted in the high bias regime. From the space charge limited model, the out-of-plane charge carrier mobility in P3HT is found to be equal to μ ≈ 2.8 × 10^–4^ cm^2^ V^–1^ s^–1^, similar to the in-plane mobility reported in previous works, while the charge carrier density is *N*_0_ ≈ 1.16 × 10^15^ cm^–3^, also in agreement with previously reported values. From the thermionic emission model, the energy barriers at the Gr/P3HT and Au/P3HT interfaces result in 0.30 eV and 0.25 eV, respectively. Based on the measured barriers heights, the energy band diagram of the vertical heterostructure is proposed under the hypothesis that P3HT is fully depleted.

## Introduction

Hybrid van der Waals heterostructures based on 2D materials and/or organic thin films are being extensively studied^1−3^ for a variety of applications encompassing field effect transistors, organic solar cells,^4−6^ photodetectors,^7,8^ vertical transistors,^9−14^ and light emitting diodes.^15,16^ Despite solid progress, developing a better understanding of the electron transport across hybrid van der Waals interfaces remains crucial to better control functionality and enhance performance. In this context, graphene is an excellent candidate as 2D electrode to contact organic thin films due to its inherent ability to form π–π stacking and van der Waals bonds.^17^ Many studies to date aimed at unraveling the physical mechanisms behind charge injection at Gr/OSC interfaces for applications in diverse fields of micro- and nanoelectronics:^18−20^ typically, graphene is used as the bottom electrode in barristors^9−14^ or transferred on top of an OSC film together with a protecting polymer, e.g., PMMA. However, other more complex multilayer designs could benefit from graphene full potential as monatomic thick, semitransparent, flexible and surface-conformal electrode. For instance, graphene could replace the base in vertical transistors, enabling organic transistors with nanoscale channels and higher operation frequencies that could meet the requirements of high-frequency applications, or function as interlayer electrode in OLEDs.^21−24^ In this framework, the development of large-scale photolithographic fabrication methods compatible with hybrid architectures that exploit graphene as the top or interlayer electrode, and the understanding and modeling of the charge transport in the latter is crucial for the design and optimization of novel functional devices.

For this study, the authors developed a fabrication process for Au/P3HT/Gr hybrid VdW heterostructures, where PMMA-free graphene lies on top of a p-type OSC and functions as the top electrode for the vertical stack, and investigated and modeled the charge injection across the two interfaces, i.e., Au/P3HT and P3HT/Gr, by temperature-dependent *I*–*V* measurements, impedance spectroscopy, and Kelvin probe force microscopy (KPFM). The charge transport across the device was found to be described by the thermionic emission (TE) assisted by image-charge induced barrier lowering model in the high voltage regime (|V| > 1 V) and by the space-charge limited (SCL) current model in the low voltage regime (|V| < 1 V). The models allowed us to extract the charge carrier concentration and the out-of-plane mobility of P3HT, and the reduced effective Richardson constant of and the potential barrier height at the two interfaces, ultimately making it possible to sketch the energy band diagram of the whole stack.

## Experimental Methods

### Materials

Poly(3-hexylthiophene-2,5-diyl) (Regio-Regular (RR) > 99%, *M*_n_ = 27 000–45 000) was purchased from Tokyo Chemicals and used as received to prepare solutions of 10 mg/mL in chlorobenzene. Graphene was grown in-house by chemical vapor deposition (CVD) on copper foils with a fully automated setup. The graphene growth protocol can be found in previously reported works.^25−27^

### Fabrication

**Figure 1:**
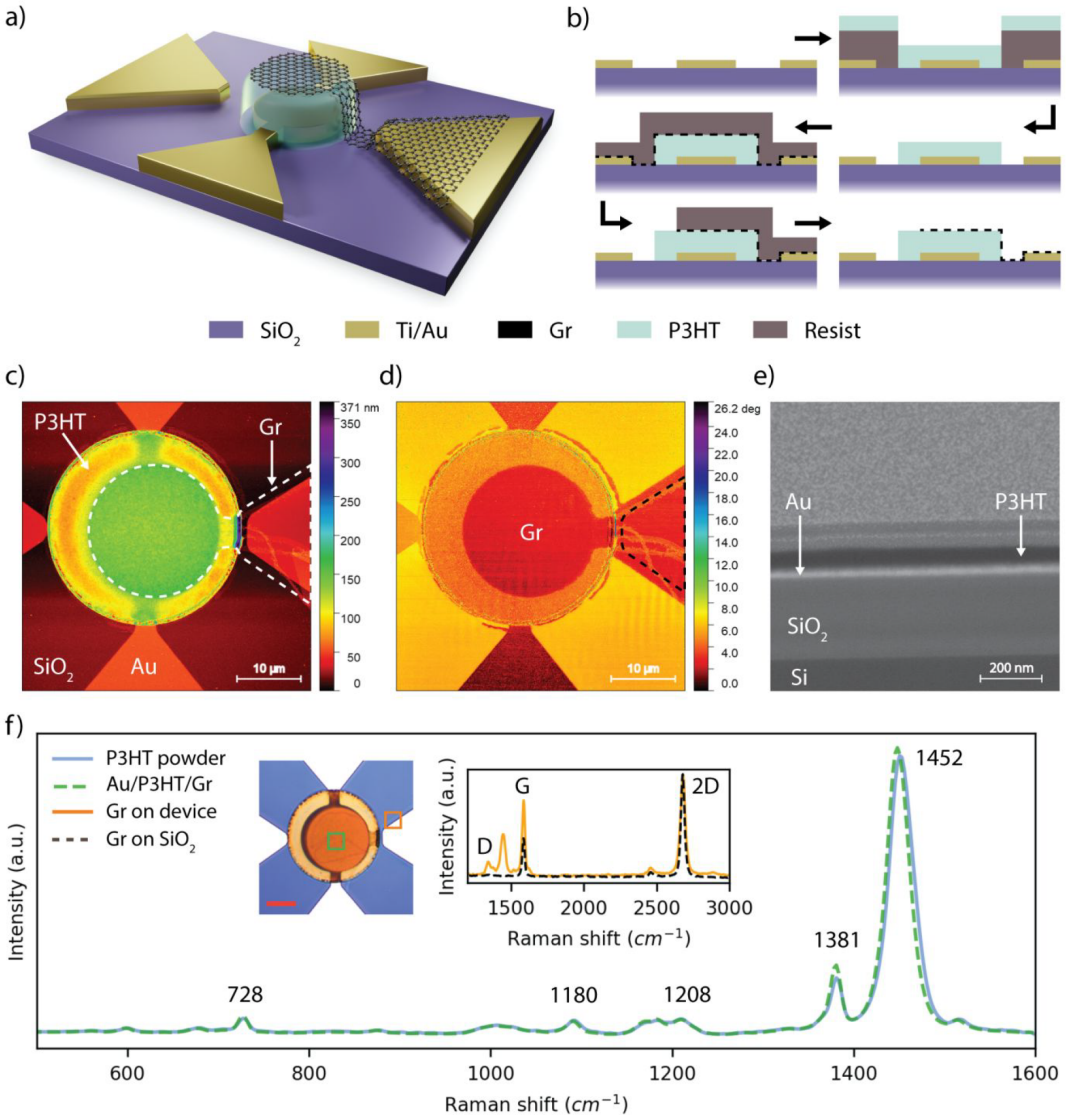
(a) 3D schematic of a representative Au/P3HT/Gr heterostructure (not to scale). (b) Schematic of the fabrication process. AFM (c) height and (d) phase images of a representative 20 μm device. (e) SEM of a FIB cut cross-section of a representative Au/P3HT/Gr heterostructure in the center of the device. (f) Raman spectra of P3HT powder (blue line) and of a representative Au/P3HT/Gr device (dashed green line). The optical image shows the acquisition position of the spectra (the red scale bar is 10 μm). The inset shows the Raman spectra of a device graphene against the Raman spectrum of a representative CVD graphene on SiO_2_.

**Figure 2:**
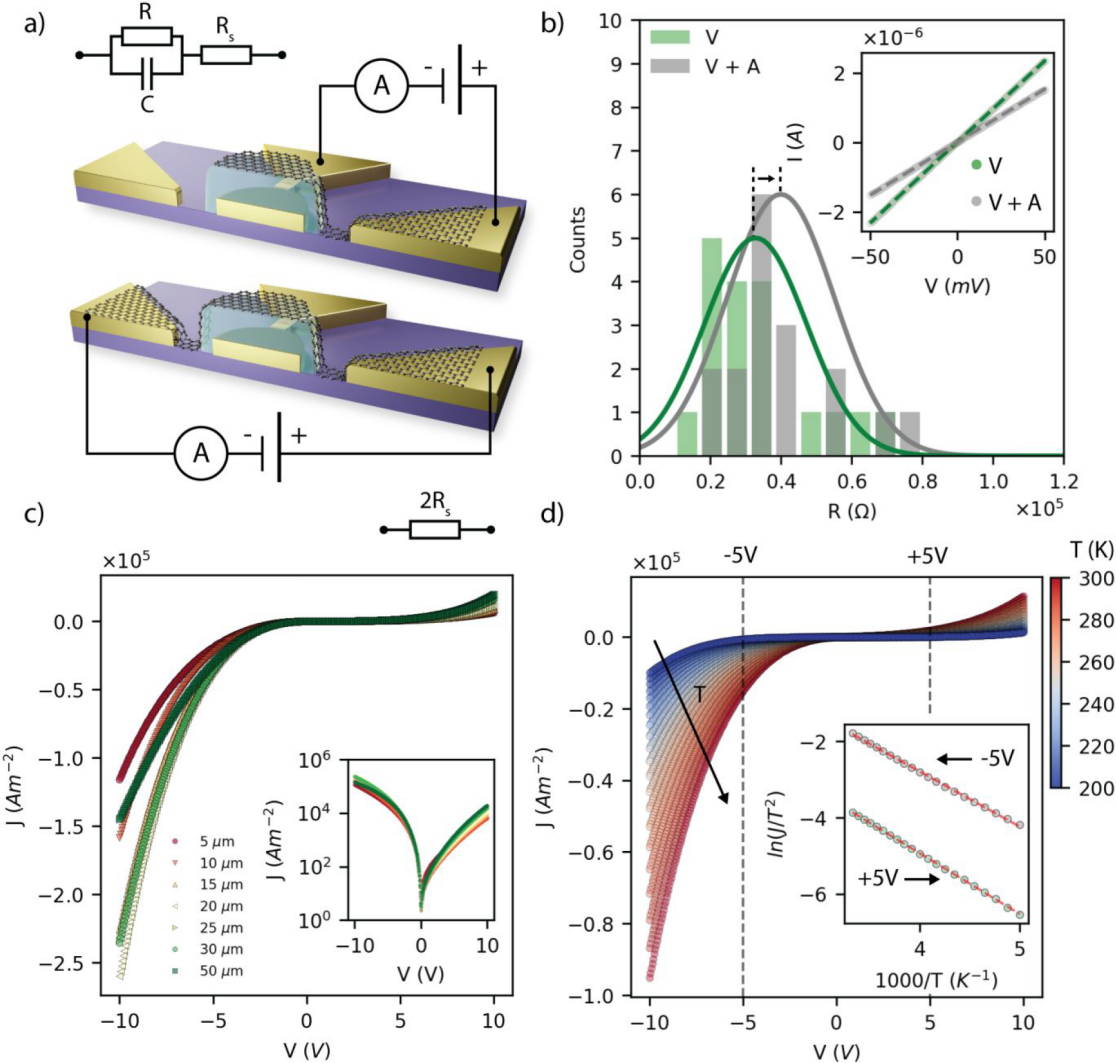
(a) Device schematics and electrical schemes of the Au/P3HT/Gr stack and of the graphene bridge devices. *R*_s_ is the graphene series resistance, *R* is the out-of-plane resistance, and *C* is the geometrical capacitance of the device. (b) Distribution of *R*_s_ in vacuum and in vacuum after annealing (17 samples). The inset shows two representative *I*–*V* traces of side-contacted graphene. The resistance is calculated from the linear fit (dashed lines). (c) Current density of representative devices with diameter 5, 10, 15, 20, 25, 30, and 50 μm. The inset shows the same traces on log scale. (d) Temperature-dependent *J*–*V* characteristic of a 5 μm device from 200 to 300 K in steps of 5 K. The inset shows the Richardson plot for 5 V and −5 V.

The study was conducted on a single chip including two different sets of devices: (i) Au/P3HT/Gr vertical stacks (119 devices) and (ii) graphene bridges (34 devices) (refer to Figure 1a and 2a for a schematic of the devices architecture). The chip was fabricated on a Si(525 μm)/SiO_2_(300 nm) substrate at the Binnig and Rohrer Nanotechnology Center (BRNC) and EMPA. In both architectures, P3HT is sandwiched between a gold (bottom) and a graphene (top) circular electrode. In the bridge architecture, graphene is side-contacted so that one can force a current through it to evaluate its resistance independently from the underlying P3HT film (see Figure 2a for the electrical scheme). The chip includes devices having various nominal diameters, i.e. 5, 10, 15, 20, 25, 30, and 50 μm. The bottom gold electrodes are 2 μm larger than the top graphene electrodes. The fabrication was done by photolithography under ambient conditions, as illustrated in Figure 1b and thoroughly described in the Supporting Information. Briefly, Au electrodes were deposited by e-beam physical vapor deposition (EBPVD) and patterned by lift-off. P3HT was then spin-coated at 1000 rpm for 60 s and patterned by lift-off. Finally, the CVD graphene top electrode was wet transferred and patterned by reactive ion etching (RIE).

### Electrical Characterization

The electrical characterization at room temperature was done in the dark, in air, under vacuum (∼1 × 10^–6^ mbar), using a Keithley 236 source-measure unit controlled via Python. The voltage was swept in the range from −10 V to +10 V in steps of 50 mV, with sweep rate of ca. 100 mV/s and internal averaging of 20 ms, keeping the bottom Au electrode on ground. The graphene resistance was characterized in graphene bridge devices by sweeping the voltage in the range from −50 mV to +50 mV in steps of 1 mV, with a sweep rate of ca. 3 mV/s and internal averaging of 20 ms.

The temperature-dependent *I*–*V* traces were collected in the range 200–300 K in steps of 5 K in a Lakeshore probe station (CRX-6.5K) operating under vacuum (∼1 × 10^–6^ mbar), in the dark. The electronics comprised an AdWin Gold II ADC-DAC unit operating at 100 kHz and a low-noise current to voltage converter (Basel SP983C). The ADC-DAC was controlled via *Python*. The voltage was swept in the range from −10 V to +10 V in steps of 0.1 V, with internal averaging of 20 ms and a delay between the source and measurement point of 100 ms, corresponding to an effective voltage sweep rate of ca. 0.8 V/s.

Impedance spectroscopy was carried out on one representative device per area using an Agilent 4294a precision impedance analyzer controlled via *Python*, from 40 Hz to 1 MHz in 201 steps, in the dark, under a vacuum (∼1 × 10^–6^ mbar), with the oscillator level set to 100 mV. Open/short compensation was performed after the acquisition and following the *Agilent Impedance Measurement Handbook*.^28^ To this purpose, we designed and fabricated devices for open/short compensation on the same chip.

KPFM measurements were carried out at room temperature in air (22 °C and 35% relative humidity) with a Dimension 3100 (Bruker), using a Pt/Ir tip. Topography (tapping mode AFM) and KPFM images were recorded using a standard two-pass procedure, in which each topography line acquired in the tapping mode is followed by the acquisition of CPD (contact potential difference between the tip and the sample) data in a lift mode. Since the CPD images on Au, Gr, and P3HT are acquired with the same tip, the interface barrier energy is directly given by the difference in the CPD values, i.e., Φ_B,Gr/P3HT_ = *q*(CPD_Gr_ – CPD_P3HT_), where *q* is the electron charge.

### Raman Spectroscopy

Raman spectra were acquired in ambient conditions using a 532 nm excitation wavelength with a WITec Alpha 300R confocal Raman microscope mounting a LD 100× objective (Zeiss EC Epiplan-Neofluar Dic, NA = 0.75) and a 300 mm lens-based spectrometer (grating: 600 g mm^–1^) equipped with a TE-cooled charge-coupled device (Andor Newton). P3HT powder and films spectra were acquired by averaging over a 5 × 5 μm^2^ area with a laser power and an integration time of 0.1 mW and 0.1 s, while graphene spectra were acquired with a laser power and an integration time of 1 mW and 10 s.

### Atomic Force Microscopy (AFM)

AFM height and phase images were collected in tapping mode in ambient conditions using a Bruker Icon AFM equipped with a TESPA-V2 cantilever with a tip apex radius of 7 nm (resonant frequency: 320 kHz, spring constant 37 N/m).

### FIB-SEM

The device cross-section was prepared by means of a FEI Helios 660 G3 UC FIB/SEM-System. Prior to cutting, a protective layer of platinum was deposited in a two-step process, first by electron-induced deposition (3 keV, 800pA), followed by ion-induced deposition (30 keV, 230pA) in order to prevent ion induced damage to the layers of interest. The cross-section was cut in a 30 kV gallium ion beam at an ion current of 47 nA. The cross-section was sequentially polished at different ion currents, down to a minimal current of 790 pA.

### Modeling, Fitting, and Plotting

Modeling, fitting, and plotting of the data were done in *Python*. Three main libraries were used (i) numpy polyfit,^29^ for the estimation of graphene series resistance; (ii) scipy curve_fit,^30^ for the SCL and TE modeling; and (iii) impedance.py,^31^ for the circuit modeling and fitting of the impedance analysis measurements.

## Results and Discussion

Figure 1a shows the schematic of a Au/P3HT/Gr heterostructure, fabricated according to the procedure illustrated in Figure 1b and described in the Experimental Methods and in the Supporting Information. Figures 1c,d show the AFM height and phase images of a representative device having a diameter of 20 μm, where a white dashed line marks the contour of the graphene and a black dashed line marks the Au side-electrode. The thickness of the Au/P3HT/Gr stack in the center of the device is ca. 130 nm as measured by AFM (see the Supporting Information). Given that the bottom Ti/Au electrode is 35 nm thick, the thickness of the P3HT film is ca. 100 nm. Figure 1e shows a cross-section of the Au/P3HT/Gr stack in the center of the device. Starting from the bottom, one can distinguish Si (525 μm), SiO_2_ (300 nm), Ti (5 nm), Au (30 nm), and P3HT (100 nm) as annotated in the figure. The graphene electrode is too thin to be visible in the cross-section. Figure 1f superimpose the Raman spectrum of the P3HT powder as received, with the Raman spectrum of the Au/P3HT/Gr stack. The vibrational modes of P3HT are found at 728, 1180, 1208, 1381, and 1452 cm^–1^, in agreement with the literature.^32,33^ The vibrational modes of graphene are not discernible from P3HT for three reasons: (i) the G and D peaks of graphene are hidden by the overlapping modes of P3HT at 1381 and 1452 cm^–1^, (ii) the 2D peak is hidden by the strong background signal of P3HT, and (iii) the P3HT is much thicker than graphene, therefore resulting in a much stronger spectral signal. Therefore, the Raman spectrum of graphene was measured on SiO_2_, in close proximity to the Au contact. The graphene Raman spectrum is shown in the inset of Figure 1f against the Raman spectrum of a representative CVD graphene on SiO_2_. The characteristic G (1580 cm^–1^) and 2D (2680 cm^–1^) peaks of graphene^34^ are identified, as well as the D (1350 cm^–1^) peak, possibly due to defects induced by the fabrication, and an additional peak at 1445 cm^–1^, most likely due to P3HT or resist residues. The AFM, SEM, and Raman data demonstrate that the fabrication process is compatible with P3HT and graphene and therefore suitable for the fabrication of vertical van der Waals devices based on these materials.

The electrical properties of OSCs are very sensitive to the environment. Figure S3 shows the *J*–*V* traces of a representative 10 μm device measured in ambient, in vacuum and in vacuum after annealing at 110° for 12 h. The current density is higher in ambient and it decreases in vacuum, reaching a minimum after annealing, with peak current density at −10 V going from 5.4 × 10^5^ Am^−2^ to 1.5 × 10^5^ A m^–2^. The traces are asymmetric: defining the rectification ratio as RR = *J*(−10*V*)/*J* (10*V*), the latter increases from RR = 1.9 in ambient, to RR = 2.6 in a vacuum, and finally to RR = 19.2 in a vacuum after annealing. This trend is ascribed to the graphene and P3HT dedoping: it is known that P3HT is doped by O_2_,^35^ while graphene is doped by O_2_ and H_2_O,^36−38^ and that their doping level can be reduced by annealing under a vacuum.^39,40^ Accordingly, an annealing under vacuum shifts the Fermi level of graphene, resulting in a realignment of the energy bands at the OSC/graphene interface, which leads to the observed change in the rectification ratio. The hypothesis is further supported by the graphene resistance shown in Figure 2b: the graphene/Au interface is Ohmic and the graphene resistance increases after vacuum exposure and annealing.

In order to minimize the variability among different devices due to uncontrolled doping of P3HT and graphene, the charge transport analysis that follows was done in a vacuum after annealing for 12 h at 110 °C. Figure 2c shows the current density of five representative devices, one per device area, measured in a vacuum after annealing (see the Supporting Information for the *J*–*V*s of all devices). The current density is calculated assuming the area of the (smaller) graphene electrode (*J* = *I*/*A*_Gr_). The current density variability falls within ca. one order of magnitude (between 7.3 × 10^4^ and 2.6 × 10^5^ at −10 V, and between 3.1 × 10^3^ and 2.3 × 10^4^ at +10 V) and all *J*–*V*s display the same shape. This suggests that the scaling of the device, from 50 μm down to 5 μm in diameter, does not affect the transport mechanism, and that the variability between devices is due to fabrication uncertainties. In all measurements conditions, and for both positive and negative bias, the current density grows exponentially with the applied voltage above a certain threshold. This trend is typically described by a variety of analytical models that allows us to extract transport parameters (e.g., charge carrier mobility and density and the energy barriers at the interfaces). Among these models are the thermionic emission (TE) assisted by image-charge-induced potential barrier lowering,^41^ the Poole–Frenkel emission (PFE),^41^ and the modified TE (MTE) for graphene/semiconductor interfaces.^42,43^ The fittings of the *J*–*V*s with the PFE model (not reported) were found to return relative dielectric permittivity of P3HT around 20–40, i.e., about 1 order of magnitude larger than what discussed in the literature.^44−46^ Therefore, the PFE model was excluded from the analysis. The hypothesis of the MTE model requires that the charge at the graphene/semiconductor interface depends on the bias. However, the capacitance measurements discussed in the following show that the organic semiconductor is fully depleted. Hence, the charge at the interface is bias independent and therefore the MTE model was not considered for the analysis that follows. The TE model has been successfully applied to metal–OSC interfaces^47−50^ and was found to be in good agreement also with the measurements of this work in the high voltage regime, that is |V| > 1 V. According to the TE model, the *J*–*V* traces shown in Figure 2c are the reverse currents of the Au/P3HT and Gr/P3HT interfaces for negative and positive bias, respectively. The reverse current reads:^41^
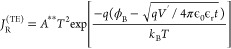
1Where A^**^ is the reduced effective Richardson constant, *T* is the temperature, *q* is the elementary charge, *ϕ*_B_ is the barrier height potential, ϵ_0_ is the vacuum permittivity, *ϵ*_r_ is the P3HT dielectric permittivity, *k*_B_ is the Boltzmann constant, *t* the thickness of the device, and *V*^*’*^ = *V* – *R*_s_*I* is the applied voltage *V* minus the voltage that drops over the graphene (series) resistance *R*_s_. The *R*_s_*I* term becomes relevant when the out-of-plane resistance of Au/P3HT/Gr is comparable to *R*_s_, which typically happens for V < −5 V and device diameter larger than 10 μm (see Figure S7).

At lower bias voltage, the *J*–*V* traces do not agree anymore with the TE model, but they show the typical trap-free space-charge limited (SCL)^41,51,52^ dependency where, on the one hand, if the charge carrier density at the contact *N*_0_ is larger than , where μ is the charge carrier mobility of the organic semiconductor, then
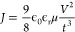
2and on the other hand, if *N*_0_ is smaller than  then
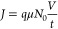
3From eqs 1–3, one can extract the barrier height *ϕ*_B_, the mobility μ, and the charge carrier density at the interfaces, provided knowledge of *ϵ*_r_ and A^**^. The effective Richardson constant A^**^ can be obtained from temperature-dependent measurements through the Richardson plot (ln(*J*/*T*^2^) vs 1/*T*), while the dielectric permittivity *ϵ*_r_ can be either taken from the literature or extracted from capacitive measurements under the hypothesis of a fully depleted semiconductor. Since the extraction of the barrier height is sensitive to *ϵ*_r_ and the latter depends on the measurement environment, it is beneficial to measure the dielectric permittivity of P3HT on the system under study, if possible. Given that the charge carrier density of unintentionally doped organic P3HT films is typically in the range of 1 × 10^17^ to 1 × 10^18^ cm^–3^,^35,40^ and that the doping concentration is usually reduced to roughly 1 × 10^15^ cm^–3^ by annealing in a vacuum,^40,53^ the P3HT can be safely assumed fully depleted and therefore *ϵ*_r_ extracted from impedance spectroscopy. This hypothesis can be assessed by measuring the capacitance of the heterostructure as a function of the applied bias: if the capacitance does not depend on the bias, then the depletion region extends over the entire thickness of the device.

A^**^ was extracted from the Richardson plot of a representative device having diameter of 5 μm at bias ±5 V, such that the graphene series resistance *R*_s_ was negligible compared to the out-of-plane resistance of the stack and therefore *V*′ (±5 V) = *V* (±5 V). Figure 2d shows the *J*–*V* characteristics as a function of temperature, from 200 to 300 K in steps of 5 K. The current density increases with temperature, peaking at −10 V from 1 × 10^4^ A m^−2^ (200 K) to 9.5 × 10^4^ A m^–2^, (300 K) while the *J*–*V*s exhibit the typical exponential character of the TE model over the whole temperature range. The inset of Figure 2d shows the Richardson plot for bias +5 V (hole injection from Gr) and bias −5 V (hole injection from Au). From the intercept of the linear fit, the reduced effective Richardson constants results in *A*_Gr/P3HT_^**^ = 4.3 A m^−2^ K^−2^ for hole injection from Gr and *A*_Au/P3HT_^**^ = 20.5 A m^−2^ K^−2^ for hole injection from Au, similar to the values previously reported for metal/OSC^47,48^ and Gr/OSC^43^ interfaces. It is worth observing that A^**^ could be extracted from the Richardson plot in the whole voltage range where the *J*–*V* is exponential and *R*_s_ is negligible. However, Figure S8 shows that (i) A^**^ does not vary significantly in that voltage range and (ii) the potential barrier heights extracted from the fittings do not depend significantly on the particular choice of A^**^. Therefore, the chosen values of A^**^ did not affect the results of this work.

**Figure 3:**
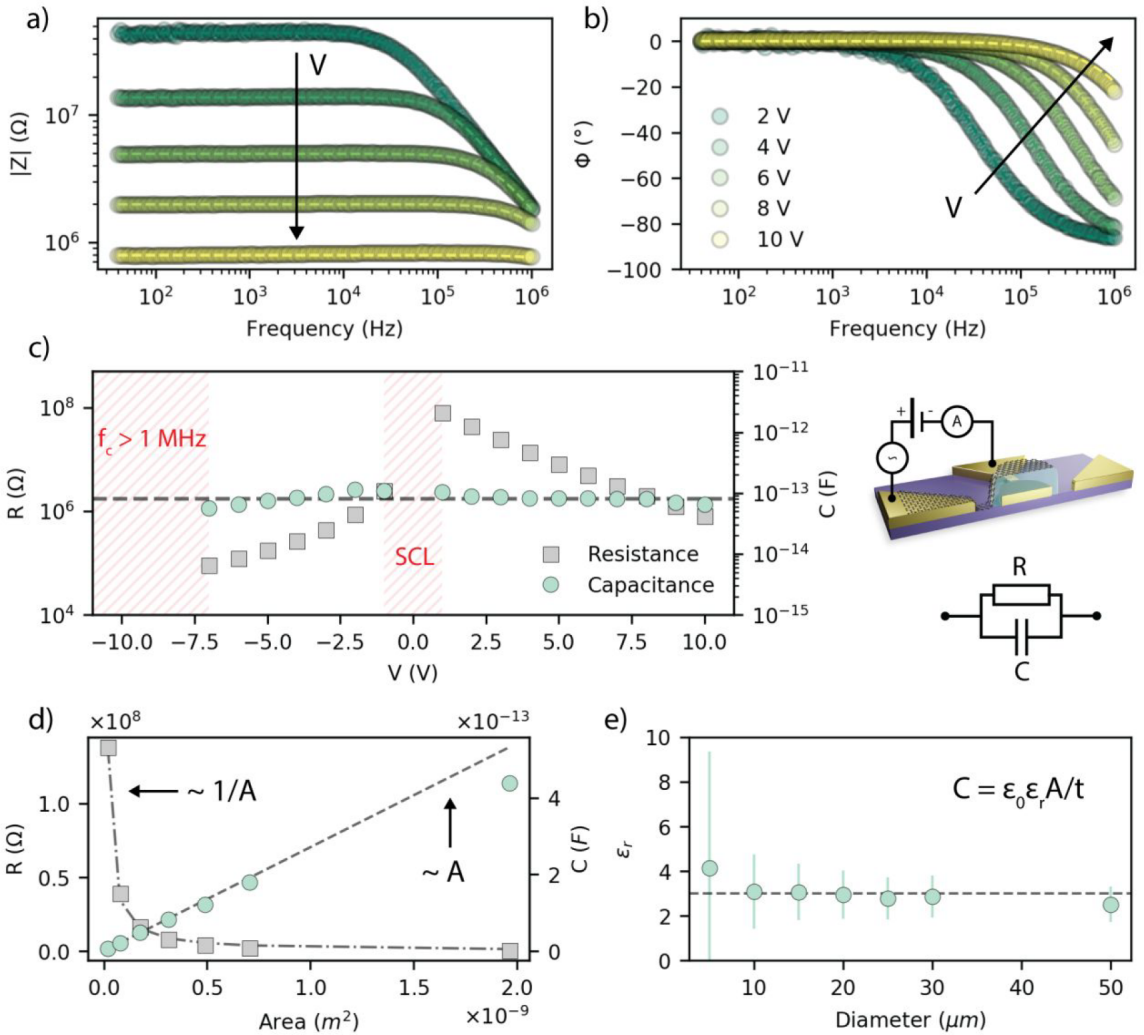
Impedance analysis. (a) Modulus and (b) phase of a representative 20 μm device: data (circles), *R*||*C* model fit (dashed lines) (c) Resistance *R* and capacitance *C* extracted from the R||C model fit at different biases. *R* and *C* are not calculated in the SCL region and for V < −7.5 V, where the cutoff frequency *f*_c_ is outside the measurement range. (d) Extracted *R* and *C* values for the devices with diameter: 5, 10, 15, 20, 25, 30, and 50 μm. The green dashed line is the linear fit of the capacitance vs area. (e) ϵ_r_ vs device diameter (error bars calculated as described in the Supporting Information).

**Figure 4:**
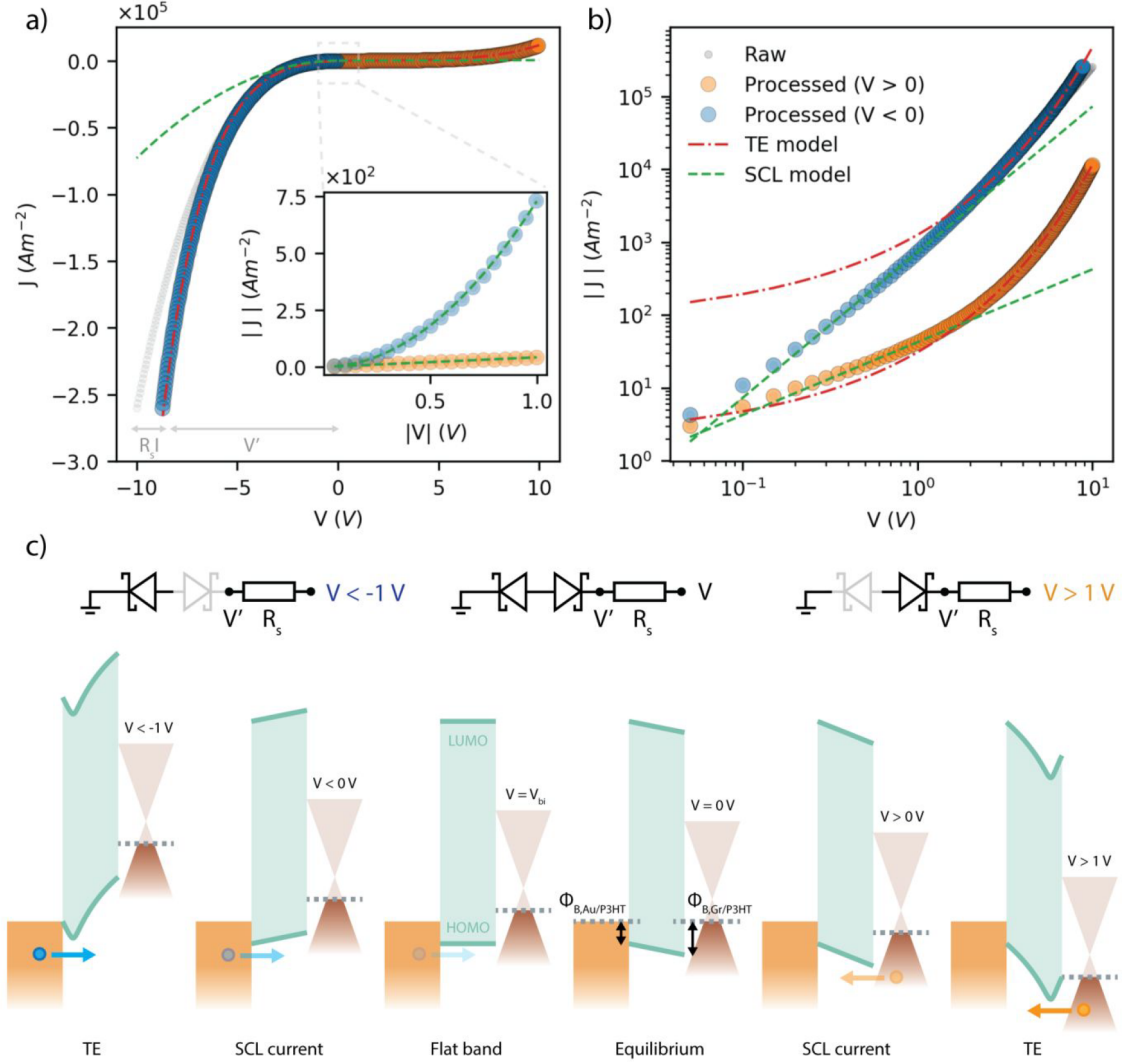
(a) Current density across a 20 μm device. Raw data are represented by gray circles. Processed data (orange and blue circles) takes into account for the graphene series resistance. The graph shows the fitting results of the SCL current (green dashed lines) and TE (red dashed lines). The inset shows the ±1 V region where the space-charge effect is limiting the current across the heterostructure. (b) Current density shown in logarithmic scale. The current density for positive and negative biases is represented by orange and blue circles, respectively. (c) Band diagram of the Au/P3HT/Gr heterojunction illustrating the charge transport regimes and equivalent circuit. The shaded Schottky diodes are forward biased.

The dielectric permittivity *ϵ*_r_ was extracted from the impedance spectroscopy on a representative device per device area. Figure 4a, b shows the impedance of a representative device having a diameter of 20 μm, in the frequency range of 40 Hz to 1 MHz, for positive bias (refer to the Supporting Information for the impedance for negative biases). The impedance exhibits the typical behavior of an *R*||*C* circuit. The resistance *R* and the capacitance *C* of the system are therefore extracted by fitting the experimental data with a nonideal capacitor model *R*||*C*, and are reported in Figure 3c. The high negative voltage range corresponding to V < −7 V was not fitted because the cutoff frequency of the system is beyond 1 MHz (upper limit of the measurement range). The low voltage region (|V| < 1 V) was also not considered because the space-charge would result in a capacitance 3 */* 2 larger than the geometrical one.^52^ The resistance decreases with the applied bias, from 80.7 MΩ at 1 V to 791 kΩ at 10 V, possibly due to the image-charge-induced potential barrier lowering, while the capacitance is bias-independent around 80 fF, confirming that the organic semiconductor is fully depleted.^54^ The dielectric constant of P3HT is estimated from the geometrical capacitance (i.e., *C* = *ϵ*_0_*ϵ*_r_*A*/*t*, where *A* is the area of the graphene electrode), without considering the edge effects and assuming a nominal thickness of 100 nm (see Figure S2), resulting in *ϵ*_r_ ≈ 3, in agreement with previously reported values for P3HT.^44−46,55^Figure 3d shows that the resistance and the capacitance scale as 1/*A* and *A*, respectively. It is worth observing that the dielectric constant calculated for small devices is affected by the large error due to geometrical variability as reported in Figure 4e.

Given A^**^ and *ϵ*_r_, one can finally use eqs 1–3 to fit the experimental *J*–*V*s and extract *ϕ*_B_, μ, and *N*_0_, as anticipated above. Figure 4a shows the *J*–*V* curve of a representative device having a diameter of 20 μm. The gray circles represent the raw data, while the orange and blue circles are the processed data for the positive and negative biases, respectively, where *V* is replaced by *V*′ = *V* – *R*_s_*I*. The barrier height and *R*_s_ are obtained by a parametric fit of the TE model (eq 1) in *R*_s_ of the processed data in the high voltage regime (|V| > 1), where *R*_s_ spans the interval 0–100 kΩ in steps of 100 Ω. The fit result in *R*_s_ = 15.4 kΩ, Φ_B,Gr/P3HT_ = 0.31 eV, and Φ_B,Au/ P3HT_ = 0.25 eV, giving a built-in potential of about 60 meV. The barrier height measured by KPFM in ambient on a representative device resulted in Φ_B,Au/P3HT_^(KPFM)^ = 0.10 ± 0.03 eV and Φ_B,Gr/P3HT_^(KPFM)^ = 0.16 ± 0.03 eV, which differ from those extracted from the fit of the *J*–*V*s, although they follow the same trend Φ_B,Gr/P3HT_ > Φ_B,Au/P3HT_ (refer to the Supporting Information for details on the KPFM measurements). This inconsistency should not be a surprise, as the KPFM strongly depends on the purity of the surface and therefore on the measuring environment,^56^ which differs from the environment of the *J*–*V* measurements. Nevertheless, the built-in potential measured by KPFM matches the value obtained from the fitting of the *J*–*V* curves. This could be ascribed to a similar shift in the graphene and gold work functions, such that the built-in potential of the stack depends mostly on the doping of P3HT when exposed to air.^35,39^ It is worth observing that Φ_B,Au/P3HT_ differs from previously reported values for Au/P3HT interfaces measured with other techniques or in different environments,^57,58^ ultimately pointing to the fact that the estimation of the barrier height is very sensitive to both the measurement conditions and the measurement method. The inset of Figure 4a shows the current density in the low voltage regime (|V| < 1). Since the built-in potential is very small, the flat-band condition is very close to the equilibrium condition. Therefore, the SCL is observed for small biases, in agreement with eq 2. Fitting the current density for negative biases with eq 2 results in an out-of-plane hole mobility of μ ≈ 2.4 × 10^–4^ cm^2^ V^–1^ s^–1^, similar to previously reported values for in-plane hole mobility in P3HT.^40,53^ Fitting the current density for positive biases with eq 3 gives the density of charge carriers at the Gr/P3HT interface, which corresponds to the intrinsic carrier concentration of P3HT (see the Supporting Information). This results in *N*_0_ ≈ 1.1 × 10^15^ cm^–3^, also in agreement with previously reported values for intrinsic P3HT in a vacuum.^40,53^ In the SCL model, the charge carrier density at the contacts depends on the density of states in the semiconductor and on the potential barrier height at the interface. From *N*_0_, one can therefore calculate the charge carrier density at the Au/P3HT interface, resulting in ∼1.2 × 10^16^ cm^–3^. The difference between *N*_0,Au/P3HT_ and *N*_0,Gr/P3HT_, is in agreement with the experimental evidence that *J* ∼ *V* for positive bias and *J* ∼ *V*^2^ for negative bias (see the Supporting Information for a discussion). The *J*–*V* dependencies are especially clear in the inset of Figure 4a and in Figure 4b.

Figure 4c shows the energy band diagram of the Au/P3HT/Gr heterostructure sketched using the barrier heights extracted from the fit of the *J*–*V*s, and assuming that the P3HT is fully depleted, as proven by capacitive measurements. The curvature of the HOMO and LUMO levels in proximity of the interfaces qualitatively describes the potential barrier lowering due to the image charge effect. The Fermi level of P3HT lies close to the HOMO level, as expected from Fermi level pinning due to interface states.^59,60^

Table 1 reports a statistical summary on five devices per area of the extracted electrical parameters. The dispersion of the extracted parameters is quite small. In order to take into account the edge effects (see the Supporting Information), the P3HT thickness of small devices was set to a value slightly larger than that measured by AFM on a representative device having a diameter of 20 μm.

**Table 1:** Statistics of the Fitting Parameters^a^

		space-charge model (|V| < 1 V)		thermionic emission model (|V| > 1 V)		
diameter (μm)	P3HT thickness (nm)	*N*_0_ (× 10^15^ cm^–3^)	μ (× 10^–4^ cm^–2^ V^–1^ s^–1^)	*R*_s_ (kΩ)	Φ_B,Gr/P3HT_ (eV)	Φ_B,Au/P3HT_ (eV)
5	130	0.94 ± 0.40	4.36 ± 0.61	35 (fixed)	0.30 ± 0.02	0.25 ± 0.01
10	120	1.14 ± 0.12	3.72 ± 0.92	35 (fixed)	0.29 ± 0.01	0.25 ± 0.01
15	100	1.11 ± 0.26	2.26 ± 0.21	42.0 ± 7.9	0.31 ± 0.01	0.26 ± 0.01
20	100	1.13 ± 0.26	2.37 ± 0.09	19.1 ± 10.9	0.31 ± 0.01	0.25 ± 0.01
25	100	1.44 ± 0.37	2.41 ± 0.21	24.6 ± 16.5	0.30 ± 0.01	0.25 ± 0.01
30	100	1.16 ± 0.26	2.13 ± 0.08	12.7 ± 6.4	0.30 ± 0.01	0.25 ± 0.01
50	100	1.20 ± 0.14	2.32 ± 0.09	11.2 ± 12.8	0.29 ± 0.01	0.25 ± 0.01
all	100	1.16 ± 0.65	2.80 ± 2.17	25.6 ± 24.3	0.30 ± 0.02	0.25 ± 0.02

a The average on five devices is given for *N*_0_, μ, *R*_s_, and Φ. The reported error is the min/max value. All SCL and TE model fits were done using *ϵ*_r_ ≈ 3, *A*_Gr/P3HT_^**^ = 4.3 A m^−2^ K^−2^ and *A*_Au/P3HT_^**^ = 20.5 A m^−2^ K^−2^. The series resistance of the 5 μm and 10 μm devices is very small compared to the device out-of-plane resistance. In order to prevent the fitting algorithm to maximize *R*_s_, the latter was set to 35 kΩ for 5 μm and 10 μm devices. Figure S5 shows the current density and the fits of various devices.

## Conclusions

This work demonstrates a potentially upscalable fabrication process for Au/P3HT/Gr VdW heterostructures on Si/SiO_2_ and describes the charge injection and transport mechanism across the heterostructures. The device output characteristic is independent from the device size for device diameters from 50 μm down to 5 μm, making device downscaling accessible and possibly limited solely by lithography resolution.

Impedance spectroscopy measurements shows that the P3HT is fully depleted in the high bias regime (|V| > 1 V or |V|/*t* > 10 MV/m), and therefore, the dielectric constant of P3HT is determined from the geometrical capacitance of the devices, resulting in *ϵ*_r_ ≈ 3. The electrical transport measurements show that the charge injection across the Au/P3HT and Gr/P3HT interfaces is dominated by TE in the high bias regime (|V| > 1 V), with potential barriers of Φ_B,Gr/*P*3HT_ = 0.31 eV and Φ_B,Au/P3HT_ = 0.25 eV, respectively, and by the SCL current in the low bias regimes (|V| < 1 V). The intrinsic carrier concentration and the out-of-plane hole mobility of P3HT, determined by fitting the *J*–*V*s in the low bias regime with the SCL model, resulted in μ ≈ 2.8 × 10^–4^ cm^2^ V^–1^ s^–1^ and *N*_0_ ≈ 1.16 × 10^15^ cm^–3^, similar to literature values extracted from in-plane FET measurements. The energy band diagram of the heterostructure shows that the interface traps/defects pin the Fermi level very close to the HOMO level of P3HT.

Since the current in Au/P3HT/Gr heterostructures is injection-limited, the hole mobility of P3HT does not limit the operating frequency of the stack, which exceeds 1 MHz for bias approaching 10 V. Higher cutoff frequencies could be achieved by making Ohmic the contact between the electrodes and P3HT, for instance, by introducing a (heavily) doped OSC layer between the electrodes and the OSC, such as F_4_TCNQ- or F_6_TCNQ-doped P3HT.

Overall, this work shows that graphene can be implemented as a top or interlayer electrode in vertical devices based on multilayer van der Waals heterostructures. For instance, the charge injection between gold and P3HT could be optimized to achieve high operating frequencies, while the Gr/P3HT interface is kept as is to exploit its rectifying nature. With graphene acting as a permeable electrode, the Gr/P3HT heterostructure studied in this work could become the core element to build future vertical organic transistors based on two back-to-back Gr/P3HT diodes.

## Supporting Information Available

Fabrication process of Au/P3HT/Gr heterostructures; FIB/SEM/AFM characterizations; electrical transport characterization, including all traces from all devices; Kelvin probe force microscopy (KPFM) analysis of the interfaces; chip overview; space-charge limited (SCL) current modeling. (PDF)

## Abbreviations

OSC: organic semiconductor
Gr: graphene
PMMA: poly(methyl methacrylate)
VdW: van der Waals
P3HT: poly(3-hexylthiophene-2,5-diyl)
TE: thermionic emission
SCL: space-charge limited
PFE: Poole–Frenkel emission
CVD: chemical vapor deposition
RR: regio-regular
OLED: organic light emitting diode
