# Supplementary material for: Charge Transport Across Au–P3HT–Graphene van der Waals Vertical Heterostructures
Source: ACS Appl Mater Interfaces. 2022 Oct 14;14(42):48240–9. doi: 10.1021/acsami.2c13148 (PMC9614726; doi:10.1021/acsami.2c13148)
Supplement: Supplementary file 1 — am2c13148_si_001.pdf [file am2c13148_si_001.pdf]

Supporting information

Charge transport across Au-P3HT-Graphene Van der Waals vertical heterostructures

Jacopo Oswald,[a,b] Davide Beretta,[a] Michael Stiefel,[a] Roman Furrer,[a] Alessia Romio,[a] Michel Daher Mansour,[d] Dominique Vuillaume,[d] Michel Calame*[a,,b,c]*

[a] Empa, Swiss Federal Laboratories for Materials Science and Technology, Transport at Nanoscale Interfaces Laboratory, Überlandstrasse 129, CH-8600, Dübendorf, Switzerland

[b] Swiss Nanoscience Institute, University of Basel, Klingelbergstrasse 82, CH-4056, Basel, Switzerland

[c] Department of Physics, University of Basel, Klingelbergstrasse 82, CH-4056, Basel, Switzerland

[d] Institute of Electronic, Microelectronic and Nanotechnology, Centre National de la Recherche Scientifique, Villeneuve d'Ascq, France.

E-mail of the corresponding authors.

Davide Beretta: davide.beretta@empa.ch

Michel Calame: michel.calame@empa.ch

Contents

Fabrication of Au/P3HT/Gr heterostructures 2
FIB/SEM/AFM Characterization 5
Electrical transport characterization 7
Kelvin Probe Force Microscopy (KPFM) 13
Chip overview 15
Space-charge limited (SCL) current modeling 16
References 19

Fabrication of Au/P3HT/Gr heterostructures

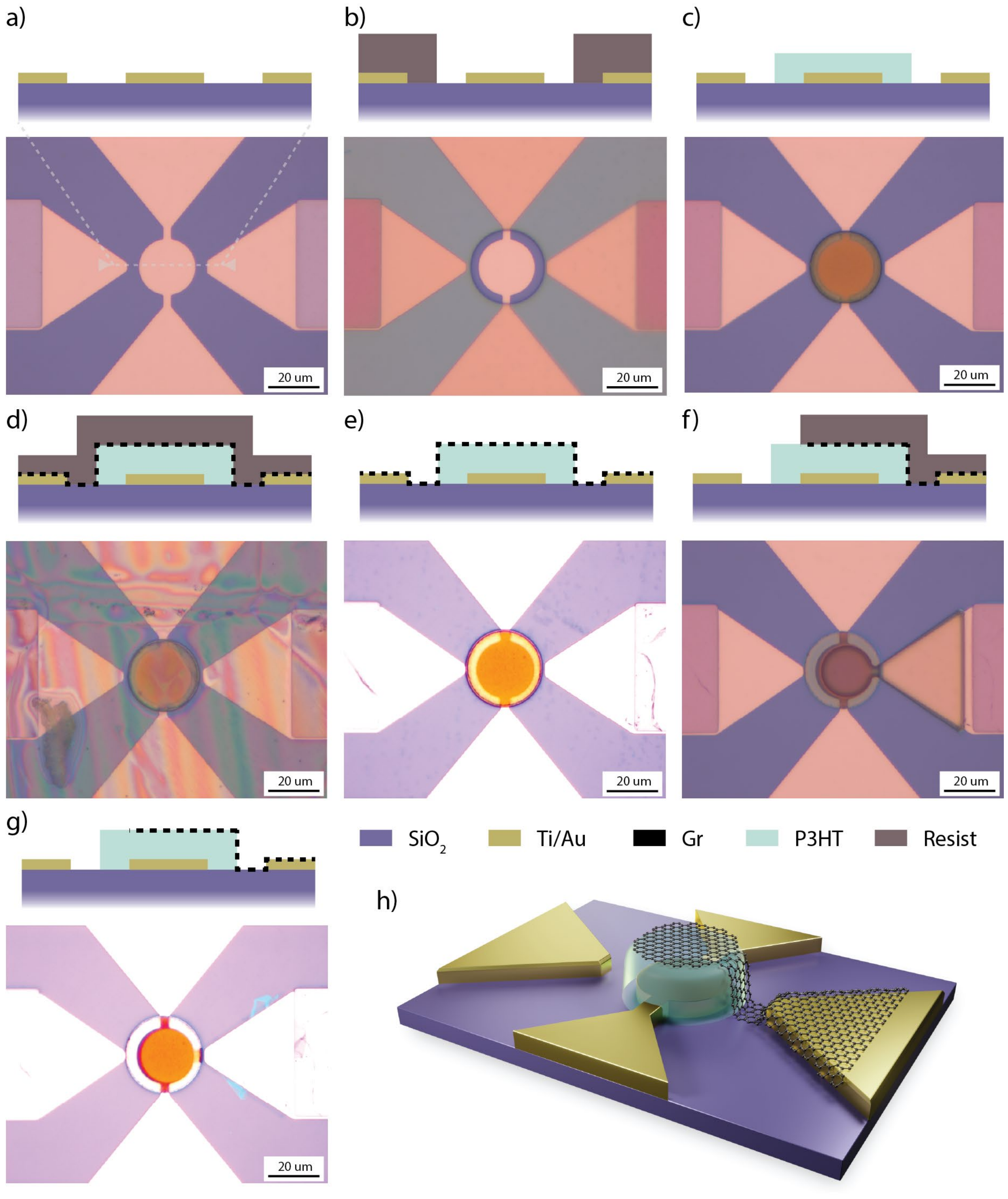

Figure S1. Fabrication steps of the vertical Au/P3HT/Gr heterostructure. (a) Patterning of the bottom electrodes. The grey dashed lines show the schematic of the section (top) on the optical microscope image (bottom). (b) Preparation of the lift-off resist. (c) P3HT deposition and

patterning. (d) CVD graphene transfer. (e) PMMA removal from graphene. (f) RIE patterning of the graphene top electrode. (g) PMMA/Optical resist removal from graphene. (h) 3D schematic of the device (not in scale)

Figure S1 shows the main fabrication steps of the Au/P3HT/Gr heterostructure, which consists in

a) *Patterning of the bottom electrodes*

   Ti (5nm) / Au (30 nm) electrodes are fabricated on a 4 inches Si (525 μm) / SiO_2 (300 nm) wafer, which is pre-cleaned in oxygen plasma (600 W for 5 min). The electrodes (Ti/Au) are deposited by e-beam physical vapour deposition (EBPVD) and patterned by lift-off in DMSO at 100°C for 30 min. The resist for the lift-off (AZ2020nlof) is spin-coated (4000 rpm for 60 s), exposed to UV light (lamp intensity 11 mW/cm^2) through an optical mask, and then developed (AZ726mif, 35 s).

b) *Preparation of the lift-off resist*

   The chip with pre-patterned electrodes (Si/SiO_2/Ti/Au) is ultra-sonicated in Acetone for 5 min, rinsed with IPA and blown dry with nitrogen. Then, it is exposed to oxygen plasma at 600 W for 5 min. After HMDS treatment, the chip is coated with a double layer positive optical resist: first, the chip is spin-coated with a LOR5B resist (4000 rpm, 40 s) and backed at 180°C for 5 min. Then, it is spin-coated with an AZ1505 positive resist (4000 rpm, 40 s) and backed at 110°C for 1 min. The device area is exposed for 1.8 s to UV light (lamp intensity 11 mW/cm^2, dose 20 mJ) through an optical mask. Finally, the exposed resist is developed in AZ400K (400K:DIW, 1:4) for 25 s and rinsed with de-ionized water.

c) *P3HT deposition and patterning*

   A 100 nm film of P3HT is obtained by spin-coating 10 mg/ml solution of P3HT in chlorobenzene (1000 rpm for 60 s) on the substrate. Subsequently, the P3HT film is patterned by lift-off in DMSO (5 min). The chip is then rinsed in de-ionized water, blown dry with nitrogen and finally annealed overnight at 110°C in vacuum (~1mbar).

d) *CVD graphene transfer*

   CVD graphene foil (Cu/Gr/PMMA) is placed to float in a copper etchant (Transene CE-100) for 1h, the PMMA layer facing upwards. Once the copper is completely etched (Gr/PMMA), the etchant is removed and replaced with de-ionized water, twice. Then, the foil is transferred to a 10% HCL cleaning solution for 5 min and transferred back to de-ionized water, twice. The

floating graphene foil (Gr/PMMA) is transferred onto the substrate (Si/SiO_2/Au/P3HT/Gr/PMMA) and let dry in air for 1 h.

e) *PMMA removal from graphene*
The chip is annealed overnight at 80°C in vacuum (~1 mbar). The top PMMA layer is removed in Acetone (5 min) and the chip annealed again overnight at 80°C in vacuum (~1 mbar).

f) *RIE patterning of the graphene top electrode*
The chip is first spin-coated with a 50K PMMA resist (AR-P 632.06, 4000 rpm for 60 s), then with an AZ1505 optical resist (4000 rpm, 40 s) and backed at 110°C for 1 min. The device area is exposed for 1.8 s to UV light (lamp intensity 11 mW/cm2) through an optical mask. The exposed optical resist is developed in AZ400K (400K:DIW, 1:4) for 15 s and rinsed with de-ionized water. Then, RIE is used to remove the first layer of PMMA 50K and graphene (O_2, 30 sccm, 25 W).

g) *PMMA/Optical resist removal from graphene*
The PMMA/Optical resist protecting the graphene electrode is removed with Acetone (1 min), then the chip is rinsed in de-ionized water and blown dry with nitrogen.

FIB/SEM/AFM Characterization

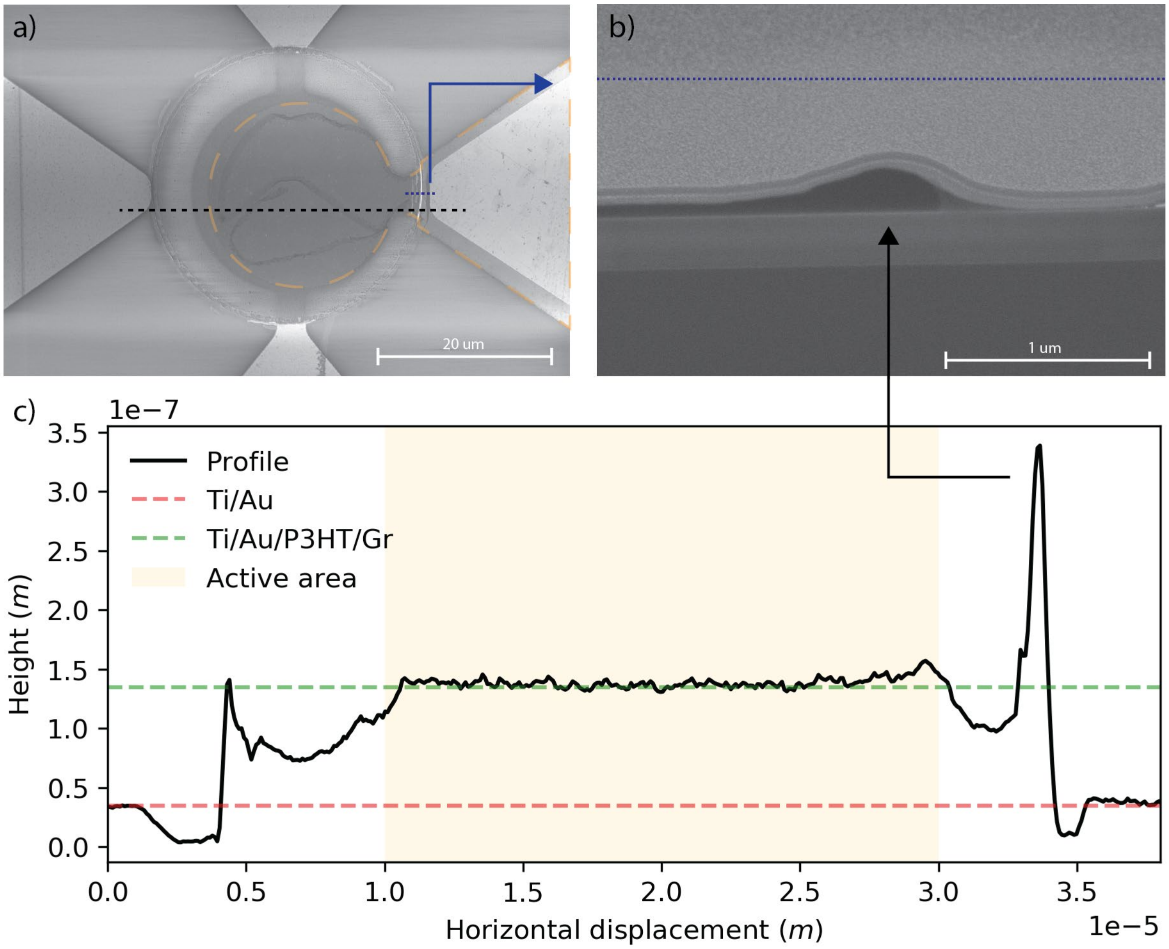

Figure S2. SEM images. (a) Top view of a representative device. The orange dashed line shows the contour of the graphene electrode. The blue dashed line shows the location of the cross-section of (b). (b) Cross-section showing the right edge of P3HT. (c) AFM height profile of the Au/P3HT/Gr stack. The red dashed line show the height of the Ti/Au electrodes, i.e. 35 nm. The green dashed line represent the height of the Tia/Au/P3HT/Gr stack, i.e. 135 nm. From this, the deduced thickness of the P3HT layer is roughly 100 nm.

Figure S2a shows the SEM image and of a representative device. The graphene electrode is clearly visible and contoured with an orange dashed line. Graphene bilayers are distinguishable on the Au side contact. Figure S2b and S2c shows the cross-section corresponding to the dotted blue and

dashed black line, respectively, in Figure S2a. The thickness of the P3HT film in the device center is uniform, while it is not on the device edge, where a higher ring possibly due to capillary/adhesion forces of the P3HT to resist prior to lift-off is observed. Although not desirable, the high edge does not affect the geometry of the device, which is entirely dictated by the region where the bottom and the top electrodes superimpose (active area shown in Figure S2c), and does represent an issue for the graphene electrode since it can easily adapt to the smooth shape of the P3HT edge (Figure S2b).

Electrical transport characterization

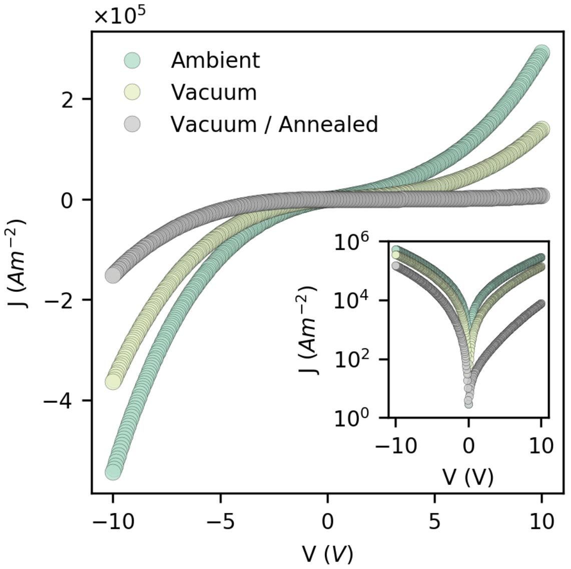

Figure S3. J-V traces of a 10 μm wide vertical Au/P3HT/Graphene device in ambient, in vacuum and in vacuum after annealing at 110 ° C for 12 h. The inset shows the same traces on log scale.

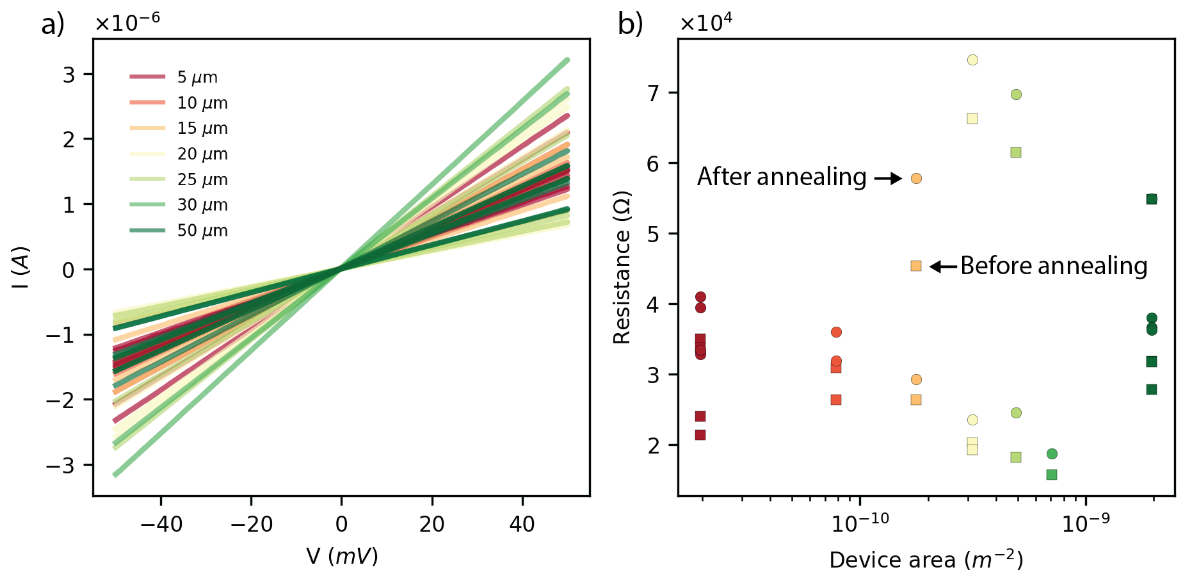

Figure S4. Graphene in-plane conductivity measurements. The total number of samples shown in the plot is 17. (a) Measured current vs. bias in the devices. Square symbols represent the devices measured in vacuum before annealing. Circles represent the devices measured after annealing. (b) Graphene resistance vs. device area.

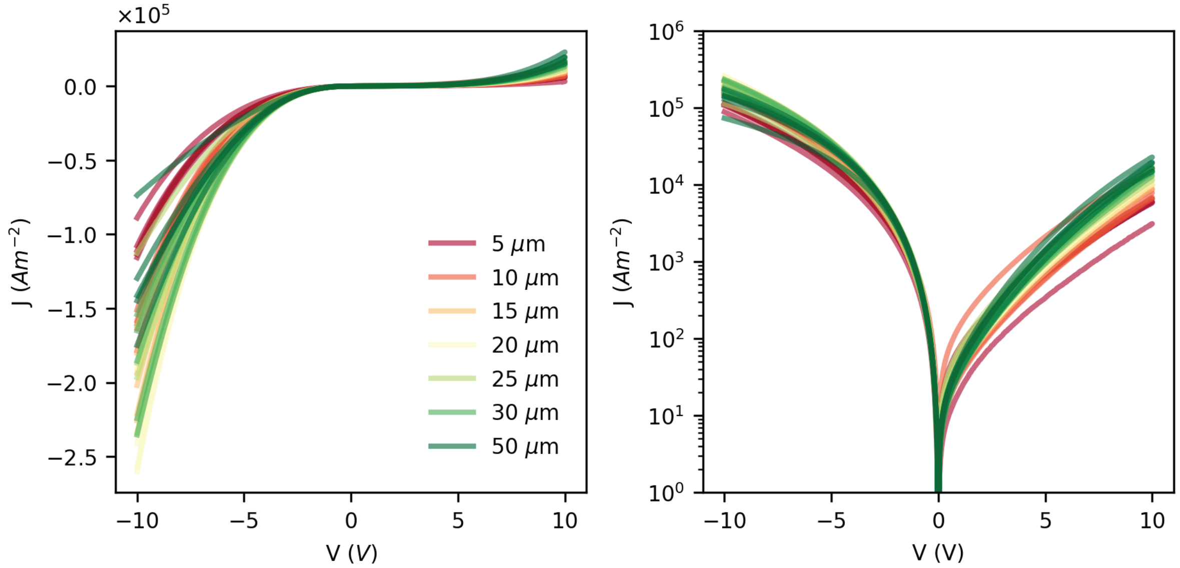

Figure S5. J-Vs of five devices per area of the vertical Au/P3HT/Gr devices measured in vacuum after annealing.

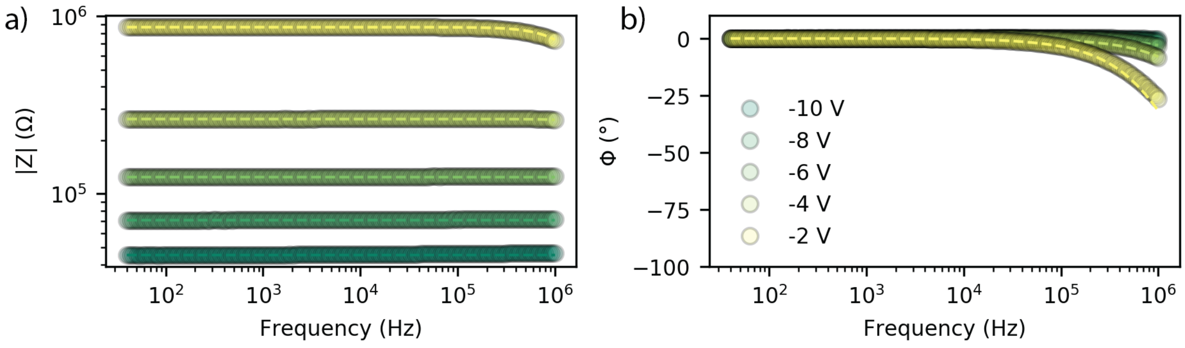

Figure S6. Impedance analysis. Modulus (a) and phase (b) of a representative 20 µm device for negative applied bias. The R||C system cut off frequency shifts above 1MHz for negative applied bias, where the resistance drops and becomes comparable to the graphene series resistance.

The dielectric constant ϵ_r of P3HT for different devices is calculated using the parallel plate capacitor equation (Eq. S1). The results are shown in Table 1.

$$C = \epsilon_0 \epsilon_r \frac{A}{t} \qquad \text{Eq. S1}$$

Where ϵ_0 is the vacuum permittivity, A is the device area and t the device thickness. The propagation errors at first order is calculates as shown in Eq. S2.

$$\Delta\epsilon_r = \frac{1}{\epsilon_0}\sqrt{\left(\frac{t}{A}\Delta C\right)^2 + \left(\frac{C}{A}\Delta t\right)^2 + \left(\frac{Ct}{A^2}\Delta A\right)^2} \qquad \text{Eq. S2}$$

Where ΔC is the fit error, Δt = 30 nm is the estimation of the thickness error and $\Delta A = \pi\ ((d_{Au} - d_{Gr})/2)^2$ is the estimation of the area error. Where d_{Au} and d_{Gr} are the diameter of the gold and graphene electrodes, respectively.

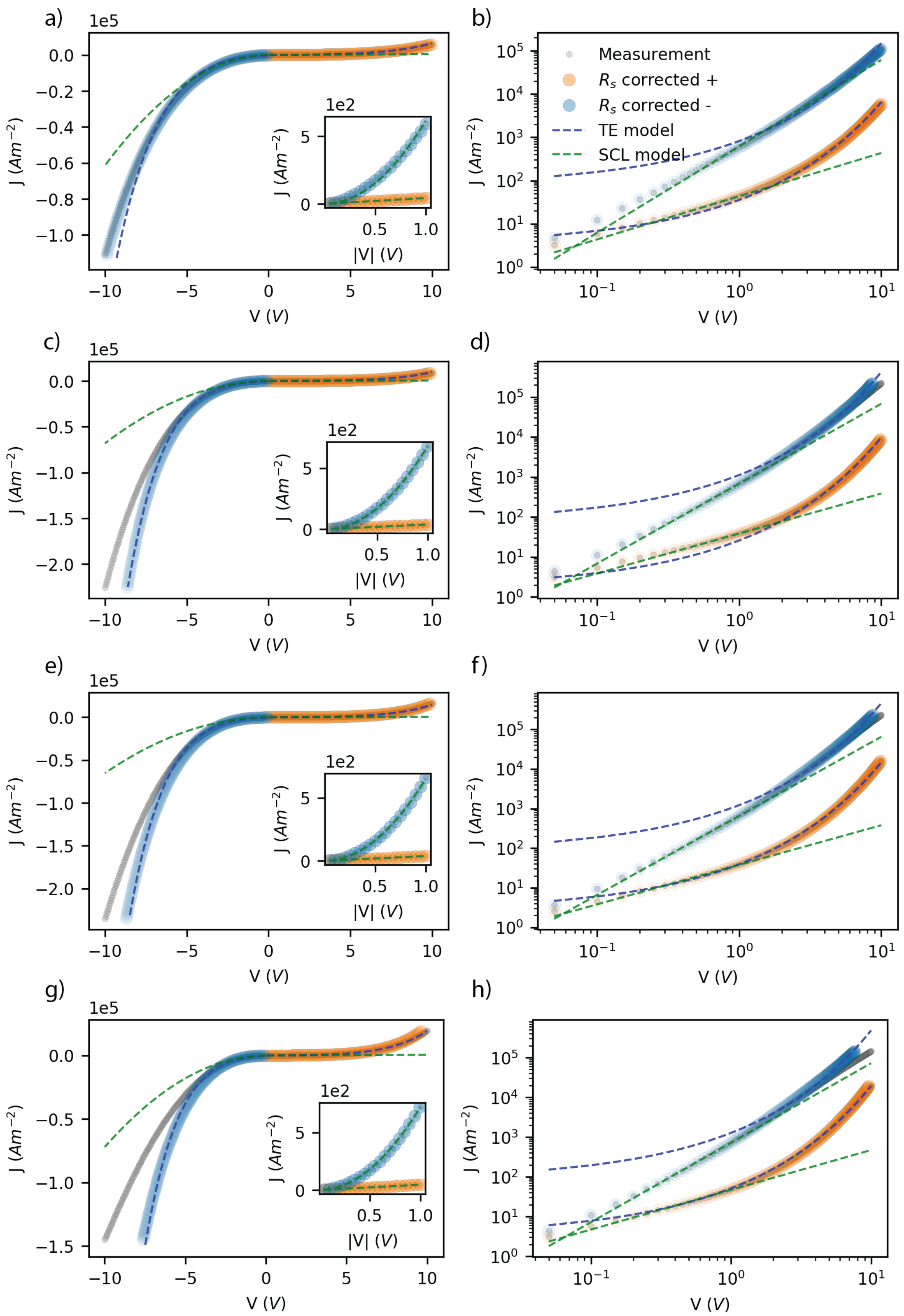

a)
b)
c)
d)
e)
f)
g)
h)
1e5
1e2
J (Am^{-2})
V (V)
|V| (V)
Measurement
R_s corrected +
R_s corrected -
TE model
SCL model

Figure S7. SCL and TE models fitting for different device with diameters: (a-b) 5 µm, (c-d) 15 µm, (e-f) 25 µm, and (g-h) 50 µm. Table 1 shows the statistic of fitting parameters. (a-b) In Fig. S2, one can observe a slightly thicker organic layer around the edge of the devices active area. The latter could have affect the actual average thickness of small devices. To take this effect into account, the thickness of the 5 µm and 10 µm device was set to 130 nm and 120 nm. For all the other devices, where the edge area can be neglected compared to the whole device area, the thickness was set to 100 nm.

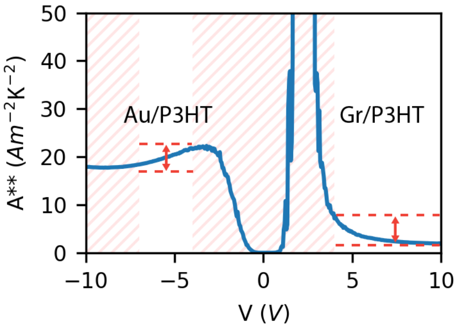

A** (Am-2K-2)		Φ (eV)	
Gr/P3HT	Au/P3HT	Gr/P3HT	Au/P3HT
2	18	0.29	0.25
2	22	0.29	0.25
7	18	0.32	0.25
7	22	0.32	0.25

Figure S8. (Left) A** as a function of voltage extracted from temperature dependent IV measurements on the representative 5 µm device. A** in the range from -10 V to -7 V is neglected because of the graphene series resistance. Similarly, A** in the range from -4 V to 4 V is also neglected because of instrumentation sensitivity. (Right) Potential barrier height calculated for A** values spanning the whole range (ca. 18-22 Am^{-2}K^{-2} for Au/P3HT and 2-7 Am^{-2}K^{-2} for Gr/P3HT).

Kelvin Probe Force Microscopy (KPFM)

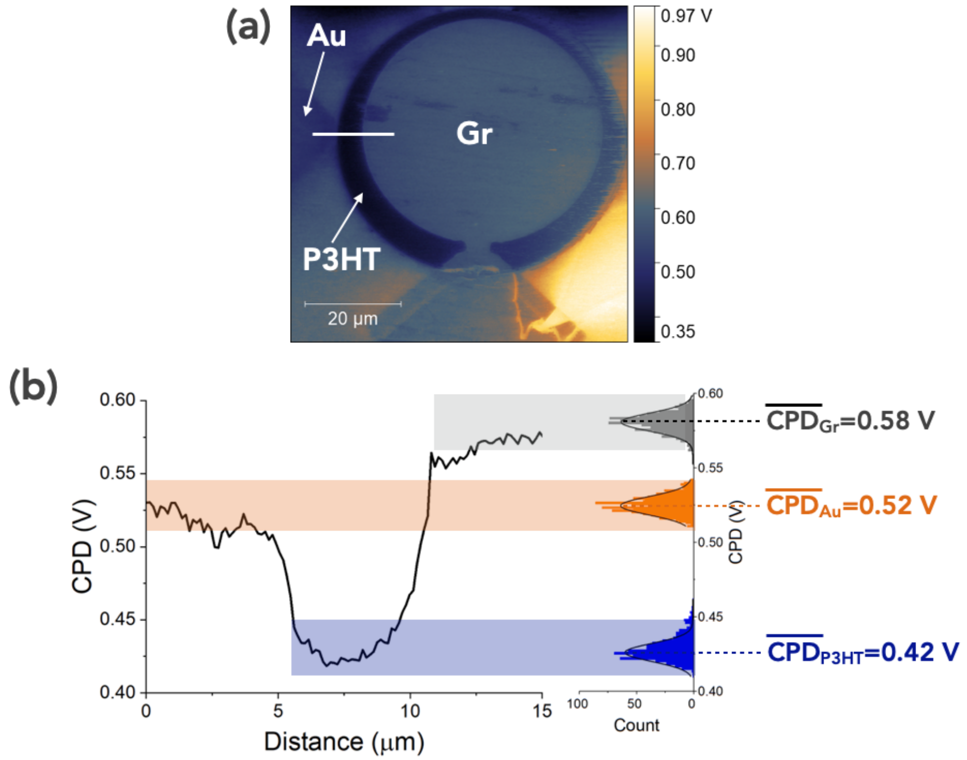

Figure S9. (a) CPD image of a 50 µm device with the different parts (bottom Au electrode, P3HT film and top graphene electrode) indicated. (b) CPD profile along the white line shown in (a) and histograms of the CPD values measured on Au, Gr and P3HT. The black lines are the fits with a Gaussian distribution, the mean CPD values are given in the figure (FWHM of 14 meV in all cases).

Figure S9a shows a Contact Potential Difference (CPD) image of a 50 µm device, where the top Gr electrode, the P3HT and the bottom Au electrode are clearly distinguishable. A CPD profile along the white line shown in Figure S9a reveals (Figure S6b) the variations of the CPD for the Au electrode, the P3HT film and the graphene electrode. CPD histograms recorded locally on the Au, P3HT and Gr are shown in the right panel of Figure S9b. Therefore, the deduced potential barrier at the interfaces are $\Phi_{B,\mathrm{Au/P3HT}}$ = 0.10 ± 0.013 eV and $\Phi_{B,\mathrm{Gr/P3HT}}$ = 0.16 ± 0.013 eV. These values show a similar trend as obtained from the I-V measurements ($\Phi_{B,\mathrm{Gr/P3HT}} > \Phi_{B,\mathrm{Au/P3HT}}$) with the same built-in potential (60 meV). However, the KPFM barrier heights are

smaller. This can be ascribed to the fact that the I-V measurements were done in vacuum after annealing. In this latter case, the obtained potential barriers at the interfaces are larger than the ones obtained from KPFM measurements done in air and ambient condition.

Chip overview

	A	B	C	D	E	F	G	H	I	J	K	GE diam.	ME diam.
A	AA	AB	AC	AD	AE	AF	AG	AH	AI	AJ	AK	5	7
B	BA	BB	BC	BD	BE	BF	BG	BH	BI	BJ	BK	5	7
C	CA	CB	CC	CD	CE	CF	CG	CH	CI	CJ	CK	5	7
D	DA	DB	DC	DD	DE	DF	DG	DH	DI	DJ	DK	10	12
E	EA	EB	EC	ED	EE	EF	EG	EH	EI	EJ	EK	10	12
F	FA	FB	FC	FD	FE	FF	FG	FH	FI	FJ	FK	15	17
G	GA	GB	GC	GD	GE	GF	GG	GH	GI	GJ	GK	15	17
H	HA	HB	HC	HD	HE	HF	HG	HH	HI	HJ	HK	20	22
I	IA	IB	IC	ID	IE	IF	IG	IH	II	IJ	IK	20	22
J	JA	JB	JC	JD	JE	JF	JG	JH	JI	JJ	JK	20	22
K	KA	KB	KC	KD	KE	KF	KG	KH	KI	KJ	KK	25	27
L	LA	LB	LC	LD	LE	LF	LG	LH	LI	LJ	LK	25	27
M	MA	MB	MC	MD	ME	MF	MG	MH	MI	MJ	MK	30	32
N	NA	NB	NC	ND	NE	NF	NG	NH	NI	NJ	NK	30	32
O	OA	OB	OC	OD	OE	OF	OG	OH	OI	OJ	OK	50	52
P	PA	PB	PC	PD	PE	PF	PG	PH	PI	PJ	PK	50	52
Q	QA	QB	QC	QD	QE	QF	QG	QH	QI	QJ	QK	50	52
Type	Open	Short	Stack	Stack	Stack.	Stack	Stack	Stack	Stack	Bridge	Bridge		

Table S1. Overview of the entire chip. Green cases show the working devices, while the red cases are the not working ones. Roughly, 50% of the chip devices are working and show the same J-V behavior of the device shown in Fig. 5.

Space-charge limited (SCL) current modeling

The analytical solution of the space-charge limited (SCL) current is here reported for convenience, as proposed in previous works.[1–4]

1) From the continuity equation:

$$J = qn\mu E(x) + qD\frac{\partial n}{\partial x} \quad \text{Eq. S3}$$

and the Poisson equation:

$$\frac{\partial}{\partial x}E(x) = -\frac{q}{\epsilon}n(x) \quad \text{Eq. S4}$$

2) Assuming that the diffusion current is negligible[3]:

$$J = qn\mu E(x) = -\epsilon\mu E(x)\frac{\partial E(x)}{\partial x} \quad \text{Eq. S5}$$

3) Integrating Eq. S5:

$$\int_0^x J d\vartheta = J\int_0^x d\vartheta = \epsilon\mu\int_0^x E(\vartheta)\frac{\partial E(\vartheta)}{\partial \vartheta}d\vartheta$$

$$Jx + K = \frac{1}{2}\epsilon\mu E(x)^2 \quad \text{Eq. S6}$$

where *K* is a constant.

4) Solving Eq. S6 for the electrical field E(x):

$$E(x) = \sqrt{\frac{2J}{\epsilon\mu}(x + K')} \quad \text{Eq. S7}$$

where $K' = \frac{K}{J}$.

5) K' is found using the boundary conditions at the injecting contact (x = 0). Defining $n|_{x=0} = N_0$ and applying the Dirichlet boundary condition $\frac{dE(x)}{dx}\Big|_{x=0} = \frac{qN_0}{\epsilon}$, K' is

$$K' = \frac{J\epsilon}{2\mu N_0^2 q^2} \quad \text{Eq. S8}$$

6) Then, plugging K' in Eq. S7:

$$E(x) = \sqrt{\frac{2J}{\epsilon\mu}\left(x + \frac{J\epsilon}{2\mu N_0^2 q^2}\right)} \quad \text{Eq. S9}$$

$$E(0) = \sqrt{\frac{J^2}{\mu^2 N_0^2 q^2}} = \frac{J}{\mu N_0 q}$$

7) Finally, the voltage associated to the current J in the semiconductor of length L is given by:

$$V = -\int_0^L E(x)dx = \sqrt{\frac{8J}{9\epsilon\mu}}\left[(L+K')^{\frac{3}{2}} + K'^{\frac{3}{2}}\right] \qquad \text{Eq. S10}$$

8) The current-voltage relation is found solving Eq. S10 for the current. Two solutions are found:

$$J = \frac{9}{8}\epsilon\mu\frac{V^2}{L^3} \qquad \text{for K'} \ll \text{L} \qquad \text{Eq. S11}$$

$$J = q\mu N_0\frac{V}{L} \qquad \text{for K'} \gg \text{L} \qquad \text{Eq. S12}$$

J is the current density driven through the device by applying the bias V. The other parameters are defined by the semiconductor properties. N_0, that it the charge carrier density at the interface, is defined by the density of states of the semiconductor N_{DOS} and by the potential barrier height Φ_B at the interface:

$$n|_{x=0} = N_0 = N_{DOS}e^{-\frac{E_{HOMO}-\Phi_M}{kT}} = N_{DOS}e^{-\frac{\Phi_B}{kT}} \qquad \text{Eq. S13}$$

The case of a 20 µm representative device shown in Fig. 5 is considered. N_0 at the Gr/P3HT interface can be measured by (i) extracting the hole mobility of P3HT using Eq. S11 for negative biases and (ii) applying Eq. S12 in the linear region for positive biases. The obtained charge carrier density at the Gr/P3HT interface is $N_0 = 1.1 \times 10^{15}$ cm^{-3}.

Then, using the potential barrier height (0.31 eV, extracted from TE model) and N_0 at the Gr/P3HT interface, N_{DOS} of P3HT can be calculated: $N_{DOS} = 2.4 \times 10^{20}$ cm^{-3}. Finally, N_0 at the Au/P3HT can be computed using Eq. S11 and the potential barrier height (0.25 eV). Obtained charge carrier density at the Au/P3HT interface is $N_0 = 1.2 \times 10^{16}$ cm^{-3}. It is worth observing that the image-charge induced lowering of potential barrier is not considered. N_0 may depend on the applied bias and be larger than the estimated value[1]. Figure S10 shows K' vs. J, i.e. the charge carrier density and the electrical field across the stack for the two different boundary conditions.

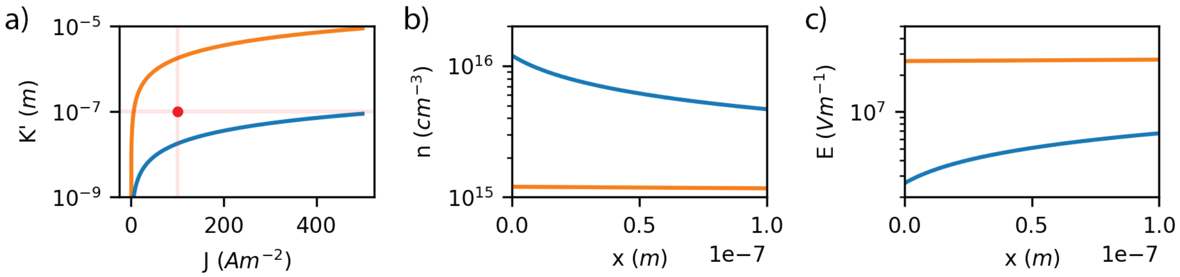

Figure S10. Space-charge limited current model calculated in the current density range of the measured devices. (a) K' vs. J showing the two different solution of the space-charge limited current model (Eq. S9 and S10). The horizontal red line shows L = 100 nm of Eq. S8. Orange line corresponds to $N_0 = 1.1 \times 10^{15}$ cm^{-3} (K' > L) and blue line to $N_0 = 1.2 \times 10^{16}$ cm^{-3} (K' < L). The vertical red line show the current density used to calculate n(x) and E(x) of plot (b) and (c). (b) Charge carrier density (b) and electrical field (c) across the across the vertical for a current density J = 100 Am^{-2}.

References

(1) Tessler, N. Experimental Techniques and the Underlying Device Physics. *J. Polym. Sci. Part B Polym. Phys.* **2014**, *52* (17), 1119–1152. https://doi.org/10.1002/polb.23550.

(2) Sze, S. M.; Ng, K. K. *Physics of Semiconductor Devices*, 3rd ed.; Wiley-Interscience: Hoboken, N.J, 2007.

(3) Schilling, R. B.; Schachter, H. Neglecting Diffusion in Space-Charge-Limited Currents. *J. Appl. Phys.* **1967**, *38* (2), 841–844. https://doi.org/10.1063/1.1709422.

(4) Mott, N. F.; Gurney, R. W. *Electronic Processes in Ionic Crystals*; Oxford University Press: London, 1940.
